# Optical Fiber Sensing Cables for Brillouin-Based Distributed Measurements

**DOI:** 10.3390/s19235172

**Published:** 2019-11-26

**Authors:** Filippo Bastianini, Raffaella Di Sante, Francesco Falcetelli, Diego Marini, Gabriele Bolognini

**Affiliations:** 1 SestoSensor S.r.l., 40069 Zola Predosa, Italy; fbi@sestosensor.com; 2 Department of Industrial Engineering-DIN, University of Bologna, 47121 Forlì, Italy; raffaella.disante@unibo.it (R.D.S.); francesco.falcetelli@unibo.it (F.F.); 3 IMM Institute, Consiglio Nazionale delle Ricerche, 40129 Bologna, Italy; marini@bo.imm.cnr.it

**Keywords:** fiber optic sensors, brillouin scattering, brillouin distributed measurements, optical fiber cables, distributed strain measurements, distributed temperature measurements

## Abstract

Brillouin distributed optical fiber sensing (Brillouin D-FOS) is a powerful technology for real-time in situ monitoring of various physical quantities, such as strain, temperature, and pressure. Compared to local or multi-point fiber optic sensing techniques, in Brillouin-based sensing, the optical fiber is interrogated along its complete length with a resolution down to decimeters and with a frequency encoding of the measure information that is not affected by changes in the optical attenuation. The fiber sensing cable plays a significant role since it must ensure a low optical loss and optimal transfer of the measured parameters for a long time and in harsh conditions, e.g., the presence of moisture, corrosion, and relevant mechanical or thermal stresses. In this paper, research and application regarding optical fiber cables for Brillouin distributed sensing are reviewed, connected, and extended. It is shown how appropriate cable design can give a significant contribution toward the successful exploitation of the Brillouin D-FOS technique.

## 1. Introduction

Brillouin-based distributed optical fiber sensors (Brillouin D-FOSs) have gained high academic and commercial interest due to their ability to provide distributed temperature and strain measurements along a several tens of kilometers long sensing fiber, with a high sensitivity and spatial resolution down to a few centimeters [1,2]. Brillouin D-FOSs are generally unmatched in fields such as the structural health monitoring of large structures, fire detection, power lines, and pipeline leakage control. Differently from conventional telecom cables designed to solely protect the fiber from damage, sensing cables used in Brillouin strain sensors must exhibit additional features in order to ensure reliable and accurate measurements. Above all, a suitable coupling between the external sheath of the cable and the sensing fiber and an optimal adhesion of the sensing cable to the structure under test are required in order to obtain a correct field reconstruction of the measured physical quantity. This paper reviews various aspects of the design and application of optical fiber cables for Brillouin D-FOS. The paper is structured as follows: Section 2 describes the operating principles of distributed Brillouin sensing with reference to the stimulated Brillouin scattering in relation to the selection of appropriate fiber cable types; issues and requirements for temperature sensing are discussed in Section 3; Section 4 provides a wide overview of aspects concerned with distributed strain measurements, such as fiber cable deployment on different substrates, theoretical and analytical models of the strain transfer mechanisms, crack detection capability, nonlinear stress transfer, overstrain protection, and the creep phenomenon in fiber sensing cables.

## 2. Distributed Brillouin Sensing

### 2.1. Stimulated Brillouin Scattering (SBS) and Brillouin Sensing in Silica Optical Fiber

**Figure 1:**
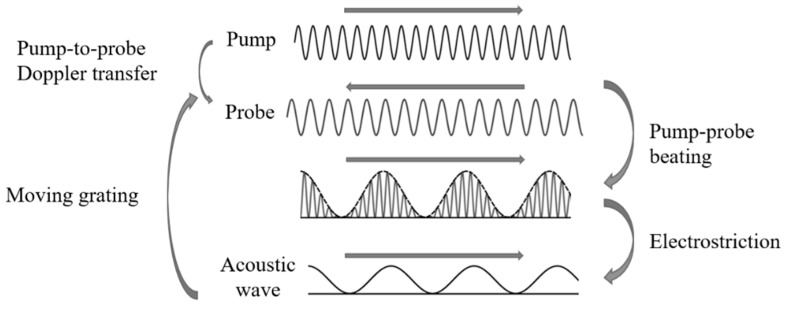
Principle of stimulated Brillouin scattering (SBS): the beating signal of the pump and probe signals generates an acoustic wave through silica electrostriction that, via modulating the material’s density and its refractive index, Doppler-transfer a fraction of pump power to the signal.

Stimulated Brillouin scattering (SBS) is an inelastic process that results from the interaction between a light wave signal, called the pump, and a red-shifted signal, called the probe, through the excitation of an acoustic wave [1]. When the two light signals travel in opposite directions along an optical fiber, the beating signal generates, due to the silica electrostriction, an acoustic wave oscillating at a frequency equal to the pump–probe frequency shift [3]. The acoustic wave perturbs the refractive index distribution of the fiber core and acts like a moving grating that Doppler-transfers a fraction of the optical energy from the pump to the probe signal (Figure 1).

The maximum probe gain is obtained when the pump–probe frequency shift is equal to the Brillouin frequency shift (BFS), $\nu_{\mathrm{SBS}}$, that depends on several physical parameters, such as strain, temperature, and pressure, and lies in the 9–11 GHz range for the most part of single mode fibers [4]. In Brillouin D-FOS, the Brillouin gain spectrum (BGS), i.e., the spectrum of SBS gain having a Lorentzian shape, is measured along the entire sensing fiber with a spatial resolution down to the cm range [5,6], enabling the estimation of the physical parameters of interest.

For a step-index fiber, the Brillouin frequency shift $\nu_{\mathrm{SBS}}$ is proportional to the silica acoustic velocity $V_{A}$ and can be expressed as [7]:

(1) $$\nu_{\mathrm{SBS}}\;=\;2\frac{nV_{A}}{\lambda_{L}},$$

where n is the refractive index of silica and $\lambda_{L}$ is the pump wavelength. The possibility to use the measurement of $\nu_{\mathrm{SBS}}$ values for the evaluation of strain, temperature, and pressure is due to the dependence of $V_{A}$ on these physical quantities. It must also be considered that other parameters, such as the fiber doping and its drawing conditions, can also significantly affect the measurement of $\nu_{\mathrm{SBS}}$, due in particular to the fact that they may vary significantly along the fiber. Consequently, they need to be taken into account in order to avoid errors in the interpretation of the experimental data. In Section 2.3, the dependence of $\nu_{\mathrm{SBS}}$ and the BGS on these parameters is described in more detail for different types of fibers (and operating conditions).

Generally, $\nu_{\mathrm{SBS}}$ also depends on the pump wavelength and this needs to be considered when comparing measurements taken with different test equipment or when replacing the interrogator equipment in an existing installation.

### 2.2. Brillouin Gain Spectrum and Brillouin Frequency Shift in Different Types of Optical Fibers

The numerous types of optical fibers available on the market show different core and cladding radii and doping profiles that result in different Brillouin gain spectra of the cables. The waveguide properties of most optical fibers are obtained by engineering the refractive index distribution of the fiber cross-section, typically through the control of the doping profile that, as described above, affects the $\nu_{\mathrm{SBS}}$ values. In particular, an increase of the core germanium (Ge) content lowers the acoustic velocity, and consequently, the Brillouin frequency shift [8]. Common telecom fibers (ITU G.652) typically have a Ge doping of 3 wt.% and a Brillouin shift around 10.845 GHz at room temperature. Ge doping is a little higher in bend-insensitive (ITU G.657) and dispersion-shifted fibers (ITU G.655 and G.653) that consequently have a Brillouin shift to a few hundred MHz lower (typically to 10.65 GHz), but it can be larger (up to 20 wt.% and more) in some specialty fibers designed for extremely low bending losses or cladding modes suppression, which consequently may have a very low (10 GHz) Brillouin shift. The Brillouin shift of some specialty fibers may be lower than, or too close to, the frequency scanning limit of some interrogators, hence preventing the possibility of using them for sensing unless an appropriate custom scanning frequency is feasible.

**Figure 2:**
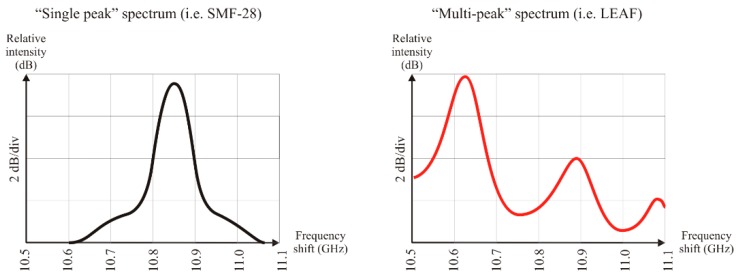
Typical “single peak” (**left**) and “multi-peak” (**right**) Brillouin gain spectra observed in SMF-28 and LEAF optical fibers, respectively.

The Brillouin gain spectrum of an optical fiber can contain one or multiple peaks depending on the acoustic modes that are excited in the SBS inside or near the core area. While most of the fibers have a single acoustic mode and their BGS exhibits a single peak, some specialty fibers, such as dispersion shift fibers with specific index profiles and/or multiple dopants, allow more acoustic modes and hence exhibit multiple peaks in the Brillouin spectrum [9,10]. Figure 2 shows the typical BGS observed in SMF-28 having a single peak (left) and the multi-peak spectrum shown by LEAF optical fibers (right). The presence of multiple peaks in the BGS of the sensing fiber can hinder the identification of the Brillouin frequency shift. In particular, if the intensity difference between two peaks is low, the simple peak search performed by the interrogator could lead to an incorrect identification of the peak due to measurement noise that randomly alters the relative heights of the peaks. In such a case, the measured temperature or strain profile exhibits multiple large “jump” errors at random positions because $\nu_{\mathrm{SBS}}$ is calculated using the incorrect peak. If the peak search is performed through a curve-fitting algorithm, the Brillouin shift could be estimated in between the two peaks if the difference between their intensity is small. In this case, it is possible to add an offset error that considers the possible shift between the actual frequency and the result frequency of the fitting process. However, such an offset error makes it difficult to compare measurements taken with different equipment or different settings of the same equipment and the error may randomly change during the sensor lifetime.

Due to this, it is generally preferable to use sensing fibers characterized by a single Brillouin peak, and when necessary, to use a fiber exhibiting multiple peaks with secondary peaks at least 5 dB weaker than the dominant one. SMF-28^®^Ultra, SMF-28^®^LL, ClearCurve^®^ XB, and LEAF^®^ (Corning Inc., Corning, NY, USA) are typical examples of multi-peak fibers that can still be used for Brillouin sensing with a negligible risk of measurement errors thanks to a good dominance (5–10 dB) of their main peak. Corning SMF-28e+^®^, at least for older production lots (before 2015), is a typical example of a dual-mode fiber having two very close (1 dB) peaks. Similar fibers should be avoided for Brillouin sensing applications even if some error mitigation techniques based on the reduction of the measurement frequency range have been proposed [11].

As described above, the Brillouin gain spectrum has a Lorentzian shape with a full width at half maximum (FWHM) value that depends on the type of optical fibers used. The FWHM value increases with the core germanium content at a rate of −94 MHz/%Ge weight. The FWHM at 1550 nm for a standard telecom fiber (Ge 3 wt.%) is typically 35 MHz whilst exceeding 65 MHz at a high germanium content (Ge 14.5 wt.%). Also, temperature and strain have been observed to affect the FWHM values: the measured temperature and strain coefficients are −0.1 MHz/°C [8] and −5 MHz/% [10], respectively, for LEAF fibers. However, some authors [8] suggest that FWHM remains constant with strain for SMF. The FWHM of the Brillouin gain spectrum influences the accuracy in the estimation of BFS values: large values of BGS bandwidth “flattens” the gain spectrum, reducing the equivalent signal-to-noise ratio (SNR) and making the peak evaluation process more difficult to perform and less accurate.

Another parameter that affects the FWHM of BGS is the temporal length of the pulsed pump that is used to interrogate the sensing fiber in pump–probe sensing schemes, such as that used in Brillouin optical time domain analysis (BOTDA) [12,13]. In particular, the measured BGS is given by the convolution of the Brillouin spectrum of the silica, having a narrow Lorentzian shape, and the spectral linewidth of the pump has a flatter Gaussian shape. This means that the smaller the temporal length of the pumped pulse, the larger its Gaussian linewidth is, and consequentially, the larger the BGS that is measured appears. Since the spatial resolution of the BOTDA measured is proportional to the pumped pulse temporal length, a relatively low (>3 m, i.e., >30 ns pump pulse) distance resolution is needed for measuring the FWHM of a sensing fiber with a reasonable (5 MHz) resolution.

### 2.3. Sensitivity of the Brillouin Frequency Shift to Temperature, Strain, Pressure, and Humidity

The values of strain, temperature, and pressure along the sensing fiber can be extracted from the Brillouin frequency shift measurements using the coefficients that relate the change of this quantity to the measurands with respect to a reference condition. The dependence of a Brillouin frequency shift on the temperature and longitudinal strain of the sensing fiber section was found to be linear in the first studies that proposed the superposition of strain and temperature effects [14]:

(2) $$\Delta \nu_{\mathrm{SBS}}\;=\;\nu_{\mathrm{SBS}}\left(\Delta T,\Delta \varepsilon \right)-\;\nu_{\mathrm{SBS},0}\;=\;\frac{d\Delta \nu_{\mathrm{SBS}}}{dT}\Delta T\;+\;\frac{d\Delta \nu_{\mathrm{SBS}}}{d\varepsilon }\Delta \varepsilon \;=\;c_{T}\Delta T\;+\;c_{\varepsilon }\Delta \varepsilon ,$$

where $c_{T}\;\approx \;1\;\mathrm{MHz}/{}^{{}^{\circ}}C$ and $c_{\varepsilon }\;\approx \;500\;\mathrm{MHz}/\%$ in SMF-28 at room temperature, and $\nu_{\mathrm{SBS},0}$ is the BFS measured for an unstrained fiber at a reference temperature. The linearity of the BFS dependence is found to be valid over a wide temperature range, −120 to 300 °C, whilst polynomial curve fittings are preferable at cryogenic temperatures [15,16]. The temperature and strain coefficients are affected by the core germanium doping (≈1.48% and ≈1.61%, respectively, per GeO_2_ unit of molar percentage) and by the draw tension applied during the fiber manufacturing process [17]. A $c_{\varepsilon }$ variation of −5% is reported for a 50% decrease of the drawing tension from the initial value of 150 g and a variation of −17% for a 100% increase. For the same changes of drawing tension, the variations of $c_{T}$ are +0.2% and +2.2%, respectively [17]. A weak cross-sensitivity has also been observed in the strain and temperature coefficients [18].

As can be seen from Equation (2), stimulated Brillouin scattering responds simultaneously to the fiber temperature and strain variations (differently from Raman scattering, which is responsive to temperature variations only [19,20]). An efficient method must therefore be established in order to discriminate the two variables. The technique commonly used is based on the use of two separated fiber cables (one is loose and subject to temperature only, the other one is adherent to the structure and is sensitive to both strain and temperature). Furthermore, the development of alternative techniques has been a subject of intense research in the last few years [21,22,23].

**Figure 3:**
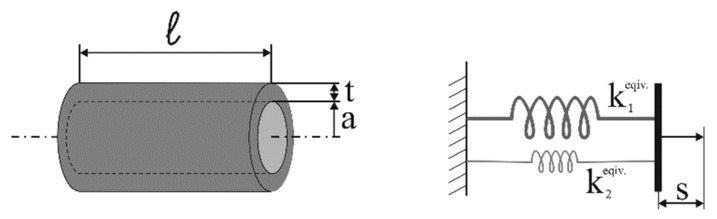
“Equivalent parallel-connected springs” model for estimating the coating influence on *c_T_*.

For a fiber embedded into a coating material, it must also be considered that the temperature coefficient is modified due to the mechanical strain that is applied to the fiber by the differential thermal expansion between the same fiber and the coating. In the case of tight mechanical coupling between the fiber and coating, the two can be modelled as parallel-connected springs (Figure 3): when a temperature change is applied, the different thermal expansions of the two materials causes stress in the fiber according to the relative stiffness between the materials. The actual fiber elongation *s* due to the differential thermal expansion can be calculated from the following equation for the general case:

(3) $$s\;=\;\frac{k_{2}^{\mathrm{equiv}.}}{k_{1}^{\mathrm{equiv}.}}l\left(\alpha_{1}\;-\;\alpha_{2}\right)\Delta T\;=\;\frac{E_{2}A_{2}}{E_{1}A_{1}\;+\;E_{2}A_{2}}l\left(\alpha_{2}\;-\;\alpha_{1}\right)\Delta T,$$

where *E* is the Young modulus, *ΔT* the temperature change, *α* the thermal expansion coefficient, *A* the cross-section, and *l* the segment length. The equivalent strain *s/l* is responsible for an additional frequency shift that can be expressed as:

(4) $$\left[\frac{\Delta \nu_{SBS}}{\Delta T}\right]\;=\;c_{\varepsilon }\frac{s}{l}\;=\;c_{\varepsilon }\frac{E_{2}A_{2}}{E_{1}A_{1}\;+\;E_{2}A_{2}}\left(\alpha_{2}\;-\;\alpha_{1}\right),$$

where *c_ε_* is the strain coefficient. The dependence of the Brillouin shift on the strain and temperature are therefore modified according to the following equation:

(5) $$\Delta \nu_{\mathrm{SBS}}\;=\;\left[c_{T}\;+\;c_{\varepsilon }\frac{E_{2}A_{2}}{E_{1}A_{1}\;+\;E_{2}A_{2}}\left(\alpha_{2}\;-\;\alpha_{1}\right)\right]\Delta T\;+\;c_{\varepsilon }\Delta \varepsilon ,$$

where *c_T_* is the temperature coefficient and *Δε* the strain change.

Quantitatively, for thin and very soft coatings, the effect on the temperature coefficient is negligible, as already reported (<0.1 MHz/°C) in the literature [24], but becomes relevant (>5%) for several coatings and materials that have wide commercial diffusion, such as Desotech DSM 9050-105, Corning CPC6^®^, copper, aluminum, Polyamide 66 (Nylon 66), etc. This also explains why the temperature coefficient of strain sensing cables is typically much different from that of the bare fibers embedded in the same cable. In the limit-case of an optical fiber embedded into a bulk substrate [25], the thermal expansion of the substrate dominates and Equation (5) degenerates into the following simplified form:

(6) $$\Delta \nu_{\mathrm{SBS}}\;=\;\left(c_{T}\;+\;c_{\varepsilon }\alpha_{substrate}\right)\Delta T\;+\;c_{\varepsilon }\Delta \varepsilon .$$

Hysteresis of the measured temperature due to the effect of the coating is also reported in the literature [26], at least for other types of optical fiber sensors, such as fiber Bragg gratings (FBGs). However, for Brillouin D-FOS, the hysteresis effect is likely to be small with respect to the other error sources already listed.

Equation (2) shows that $\nu_{\mathrm{SBS}}$ is mainly related to the axial strain along the fiber. However, Brillouin scattering is also affected by the presence of radial strain and can therefore be used to measure the hydrostatic pressure applied on the cable. Pressure coefficients, $c_{P}\;=\;\frac{d\nu_{\mathrm{SBS}}}{dP}$ of −0.91 MHz/MPa have been measured at a pump wavelength of 1550 nm in the range of 0.1–25 MPa [27,28]. Further studies that have been carried out using a pump signal of 1552.3 nm and in the pressure range of 0.1–30 MPa reported $c_{P}$ coefficients of −0.752 MHz/MPa and justified the slight change by the different coatings used as the same pressure can induce different strains in the fiber core if coatings of different types are used [29]. Furthermore, the pressure sensitivity of the values was observed to drop to −0.412 MHz/MPa when the fiber is pre-tensioned before applying the pressure increase [29].

Approximating the optical fiber to an infinite solid cylinder, the dependence of the BFS values on the axial strain Δε and pressure $p_{o}$ can be expressed with the following formula [26]:

(7) $$\Delta \nu_{\mathrm{SBS}}\;=\;c_{T}\Delta T\;+\;\left(\upsilon_{silica}c_{\varepsilon ,radial}\;+\;c_{\varepsilon ,axial}\right)\Delta \varepsilon_{axial}\;+\;\frac{c_{\varepsilon ,radial}}{E_{\mathrm{silica}}}p_{o},$$

where $\upsilon_{silica\;}\;$= 0.17 is the Poisson’s ratio for SiO_2_ glass, $c_{\varepsilon ,radial}\;$= 290 MHz/% and $c_{\varepsilon ,axial}\;$= 530 MHz/% are the radial and axial strain BFS coefficients, respectively, and $E_{\mathrm{silica}}\;$ is the Young’s modulus of silica. For coated fibers, the pressure sensitivity is influenced by the coating thickness and elastic properties, and sensitivity can be increased by using a thicker soft coating, especially when it is characterized by a low Poisson’s ratio [30].

A significant nonlinear sensitivity of the BFS value on relative humidity variation (Rh) was also observed [31] and must be taken into account because it can cause, in principle, a significant error for common variations of the environmental humidity. In particular, a BFS sensitivity to moisture variation of <0.025 MHz/%Rh is reported, as well as a decreasing influence at increasing humidity levels [31]. This effect is likely to stem from the swelling of the fiber coating due to the absorption of moisture, as is known for the polymer coating of fiber Bragg gratings and can be suppressed using an optical fiber with a seamless sealed protection, such as those used in most commercial telecom cables for outdoor use and all the cables specifically designed for Brillouin temperature sensing.

## 3. Temperature Sensing

As mentioned in Section 2.3, cables for temperature sensing are characterized by a loose mechanical coupling between the fiber(s) and their sheath in order to prevent the transfer of mechanical strain and limit the Brillouin sensitivity to temperature only.

### 3.1. Loose-Fiber Mechanical Coupling

The fiber is typically wet-coupled to the containing tubing using specific silicone gels that have the scope of improving the water resistance and lubricating the fiber-sheath coupling, thus reducing the possible stress transfer due to friction. At low temperatures, the viscosity of the used gel may, however, increase enough to allow for some drag transfer of the applied strain. This might happen when the cable is not specifically designed for pure temperature sensing.

### 3.2. Over-Length

A key element for proper cable design is fiber over-length, i.e., the fiber inside the cable is actually a little longer than the final cable length in order to ensure that no strain is applied to the sensing element, even in the case of the cable actually being subjected to some elongation up to the over-length value. Over-length must be considered for correct identification of the correspondence between the temperature/length distribution measured by the interrogator and the actual location of the measured temperature distribution along the monitored element. A typical 1% over-length means that, for a 1 km cable segment, the fiber embedded inside is 1010 m long, such that the difference between the measured strain profile and the real position along the cable is null at the beginning of the cable but becomes 10 m at its end.

### 3.3. Thermal Conductivity of the Cable and Distance Resolution Limit

The cable structure, and in particular, its axial thermal conductivity, influences how a singularity of the temperature profile is transferred from the environment to the sensing fiber, introducing some measurement distortion. In particular, a small “hot spot” (i.e., oil leakage) on the external surface of the cable, due to the combined effect of the strengthening members with high thermal conductivity and thick protective external layers with low conductivity, may result in a degree of spread over a long length of the inner sensing fiber.

**Figure 4:**
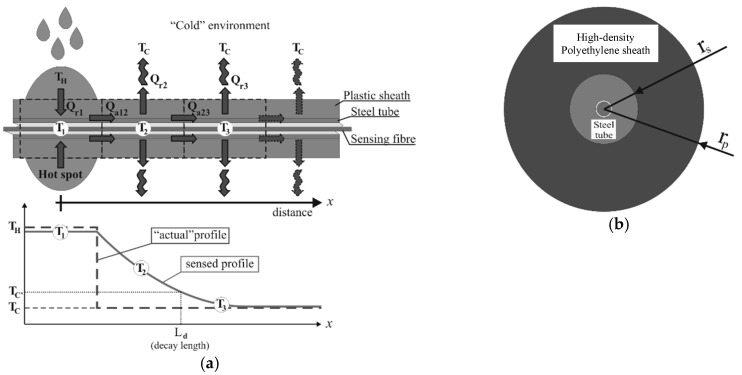
Simplified thermal model: (**a**) decay of the sensed temperature with distance in the case of a “hot spot” singularity and (**b**) schematic section of a typical armored sensing cable.

The distortion can be evaluated using the proposed simplified model depicted in Figure 4 for the thermal exchange between the cable, the hot spot, and the colder environment that surrounds the cable outside the hot spot.

**Figure 5:**
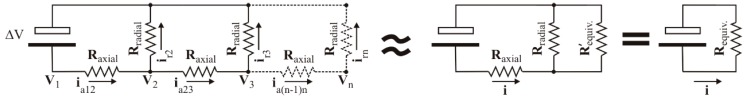
“Infinite ladder” equivalent electrical circuit model (**left**) and its classical solution (**right**).

The thermal exchange in the cable can be modelled in terms of an equivalent electrical circuit (Figure 5), in which the temperatures (*T*) are represented in terms of electrical potential (*V*), the heat flows (*Q*) in terms of currents (*i*), and the radial and axial thermal resistances in terms of electrical resistance (*R*_radial_ and *R*_axial_). The fact that the sequence of the current cells in the ladder is infinite allows us to hypothesize an equivalence between the resistances *R*_equiv_ and *R*′_equiv_, leading to the solution of the following expression [32] for the total equivalent resistance:

(8) $$R_{\mathrm{equiv}}\;=\;\frac{R_{\mathrm{axial}}\;+\;\sqrt{R_{\mathrm{axial}}^{2}\;+\;4\;\cdot \;R_{\mathrm{axial}}\;\cdot \;R_{\mathrm{radial}}}}{2},$$

where:

(9) $$\begin{array}{c} R_{\mathrm{axial}}\;=\;\rho_{\mathrm{axial}}\;\cdot \;dx,\\ R_{\mathrm{radial}}\;=\;\frac{K_{\mathrm{radial}}}{dx}, \end{array}$$

are the axial and radial thermal resistances, respectively.

Passing from the discretized (“ladder”) model to the continuum, the potential and current at the discrete points of the sensing fiber (*V_1_*, *V_2_*, …) can be expressed as a function *V*(*x*) and *i*(*x*) of the distance *x* from the hot spot, and by applying Ohm’s law *V*(*x*) *= R i*(*x*), it is possible to calculate the infinitesimal change of the potential along the cable length using:

(10) $$V\left(x\;+\;dx\right)\;-\;V\left(x\right)\;=\;-\frac{2V\left(x\right)\rho_{\mathrm{axial}}}{\rho_{\mathrm{axial}}dx\;+\;\sqrt{\rho_{\mathrm{axial}}^{2}dx^{2}\;+\;4\rho_{\mathrm{axial}}K_{\mathrm{radial}}}}.$$

The limit for d*x* 0 of Equation (10) yields the following differential equation:

(11) $$\frac{\mathrm{dV}\left(x\right)}{dx}\;=\;-\frac{\sqrt{\rho_{\mathrm{axial}}}}{\sqrt{K_{\mathrm{radial}}}}V\left(x\right),$$

the solution of which is:

(12) $$V\left(x\right)\;=\;V_{0\;}\exp\;\left(-\frac{\sqrt{\rho_{\mathrm{axial}}}}{\sqrt{K_{\mathrm{radial}}}}x\right).$$

By exploiting the model equivalence between the temperature and potential and between electrical and thermal resistances, Equation (12) can be rewritten, where the analytical solution for the temperature decay with the distance from the hot spot is given by:

(13) $$T\left(x\right)\;=\;\left(T_{H}-T_{C}\right)\;\exp\;\left(-\frac{\sqrt{\rho_{\mathrm{axial}}}}{\sqrt{K_{\mathrm{radial}}}}\;x\right)\;+\;T_{c},$$

where *T_H_* is the hot spot temperature, *Tc* is the colder environment temperature, and *x* is the distance from the hot spot.

From Equation (13), it is clear that the decay speed of the temperature profile sensed by the optical fiber depends on (*P*_axial_*/K*_radial_)^½^, which includes the effects of the cable geometry, thermal conductivity of the cable materials, and convective heat exchange between the cable and the colder environment.

For a typical armored sensing cable (Figure 4b), the axial and radial specific thermal resistivities can be respectively approximated as the parallel connection of the axial thermal conduction resistance of the plastic sheath and of the steel armor and as the series connection of the radial thermal conduction resistance of the plastic sheath with the natural air convection to the environment according to:

(14) $$\begin{array}{c} \rho_{\mathrm{axial}}\;=\;\frac{1}{k_{p}A_{p}\;+\;k_{s}A_{s}}\;=\;\frac{1}{\pi }\frac{1}{k_{p}\left(r_{p}\;-\;r_{s}\right)+\;k_{s}\left(r_{s}-\;r_{h}\right)},\\ K_{\mathrm{radial}}\;=\;\frac{hr_{p}\left(r_{p}\;-\;r_{s}\right)+\;k_{p}\left(r_{s}\;+\;\frac{r_{p}\;-\;r_{s}}{2}\right)}{2\pi hk_{p}r_{p}\left(r_{s}\;+\;\frac{r_{p}\;-\;r_{s}}{2}\right)}, \end{array}$$

where *k_p_* is the thermal conductivity of the plastic, *k_s_* is the thermal conductivity of the steel, *r_p_* is the outer radius of the plastic coating, *r_s_* is the outer radius of the steel tube, *r_h_* is the inner radius of the steel tube, *h* is the convection coefficient (colder environment), *A_p_* is the area of the plastic element in the section, and *A_s_* is the area of the steel element in the section.

As an example, considering a high-density polyethylene (HDPE)-coated temperature sensing cable with *r_p_* = 3.5 mm, *r_s_* = 1.5 mm, and *r_h_* = 0.5 mm; considering an HPDE material for the cable sheath with *k_p_* = 0.45 W/(m∙K), stainless steel for the tube with *k_p_* = 45 W/(m∙K), and *h* = 2 W/(m^2^∙K) for the natural convection cooling; and in a typical crude oil leak sensing application (*T_H_* = 80 °C, *T_C_* = 25 °C), a decay length of *L_d_* ≈ 30 cm is needed to find a temperature 97% colder than the hot spot peak. The key parameter controlling the decay speed is the axial conductivity of the cable, so increasing the section of the metal armor would increase the spread effect.

Such a peak spread is not to be necessarily regarded as a negative point, since the distance resolution limit of Brillouin interrogators may prevent the detection of very concentrated temperature changes, and the “spreading” effect increases the chance of detecting “hot spots,” even below the distance resolution limit of the interrogator equipment.

### 3.4. Use of Standard Telecom Fibers for Temperature Sensing

Apart from the controlled over-length, the structure of a standard outdoor telecom cable is not very far from that of a cable specifically designed for Brillouin temperature D-FOS. Due to this, in some cases, it is possible to use cheaper telecom cables for sensing purposes; however, care must be taken to ensure that:

- the temperature range of the application falls within the operating temperature range of the used cable;
- the cable is installed in a way that ensures that no strain is applied on the cable;
- the possible fiber length location error due to the unknown over-length is tolerable for the application.

## 4. Strain Sensing

In order to sense the deformation of the element under testing, the sensing cable must be glued or fixed to the test structure and a tight mechanical coupling is strictly needed between the external sheath of the cable and the sensing fiber. The addition of proactive coatings is an efficient solution to enhancing the fiber resistance, decreasing the probability of brittle fracture phenomena and absorbing potentially dangerous vibrations. Fiber coatings and bonding layers are usually characterized by a relatively low Young’s modulus compared with that of the fiber core. They improve the fiber resistance considerably over its operational period, but at the same time, require that the strain transfer issue from the host material toward the fiber core is carefully addressed.

Such tight coupling represents a key difference from conventional telecom cables in which the design is aimed at protecting the fibers by mechanically insulating them from the sheath; for this reason, strain-sensing applications always require specific solutions. Additionally, due to such tight coupling, strain-sensing cables may require specific handling care to avoid fiber damage during manufacturing, stocking, and installation.

In Section 4.1, issues relating to the sensing cables’ deployment on different substrates are addressed, with emphasis on the strain transfer and cable protection under specific operating conditions. Section 4.2 provides an overview of the theoretical and analytical aspects of the mechanisms regulating the strain transfer, whilst Section 4.3 analyses these aspects with respect to the crack detection capability. Nonlinear stress transfer and overstrain protection are treated in Section 4.4. Finally, Section 4.5 provides insight into the creep phenomenon that can occur in fiber cables.

### 4.1. Installation on Different Substrates

Typically, fiber cables for distributed sensing are surface-mounted or embedded in the substrate material. These techniques exhibit different features and their selection depends on the substrate characteristics, as well as on the environmental conditions. The following sub-sections give an overview of these aspects.

#### 4.1.1. Surface Installation

One key element of the surface installation is the choice between continuous or discrete mechanical coupling. In the case of continuous coupling, the sensor is tightly connected to the substrate along its entire length: this allows for the transfer of both compressive and tensile strain from the substrate to the sensor, and therefore measurement of the strain distribution in the substrate. Since continuous coupling requires no pre-stressing of the fiber, the tensile strain can theoretically be measured up to the actual failure. For this reason, it is recommended when high tensile strain is expected.

**Figure 6:**
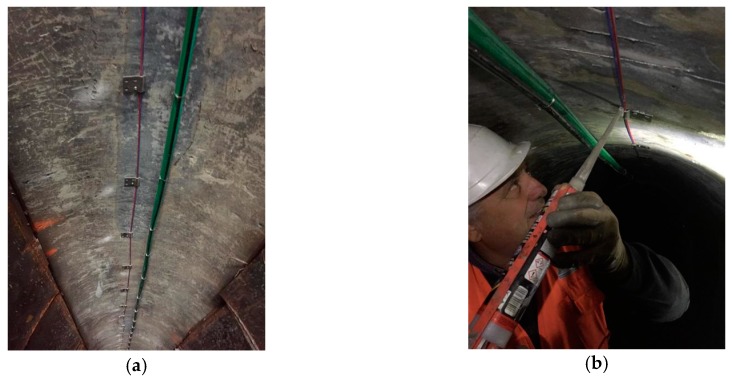
Surface installation: (**a**) sensing cable clamped at discrete locations and (**b**) glue injection in the clamps.

In the case of discrete coupling (see Figure 6), the sensor is connected to the substrate only at small spots that are equally spaced from two to five times the distance resolution of the equipment used for sensor interrogation [33]. The discretization on one side makes the resolution worse, but on the other side, averages any concentrated strain (i.e., crack) over a length long enough to allow a less sensitive but more precise quantitative measure of the actual strain. Since the fiber is suspended between two coupling points, in principle, the compressive strain of the substrate cannot be measured due to Euler instabilities related to the tiny fiber cross-section. In order to also be capable of measuring the compressive strain, it is necessary to apply a suitable pre-strain to the fiber (typically 130% of the maximum expected compressive strain) before fixing the coupling points. This, however, reduces the maximum tensile strain that can be measured before failure of the fiber.

**Figure 7:**
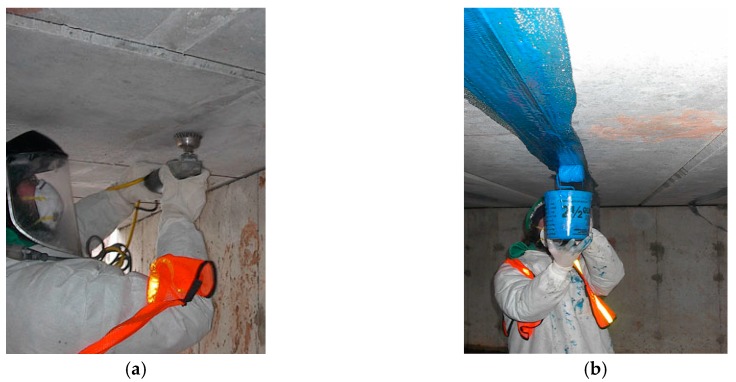
Surface installation: (**a**) preparation of the surface (mechanical grinding) and (**b**) application of the adhesive on a pre-installed sensing cable.

Surface installation is very common for steel structures and for retrofitting existing concrete members. It can be realized using adhesives or special clamps in the case of discretized connection points. The selection of the adhesive is often a key issue since it must be compatible with both the substrate and the outer sheath of the cable. Materials such as polyethylene (PE) and polyvinylchloride (PVC) require specific adhesives or primers in order to establish a good mechanical coupling. In addition, the mechanical properties of the adhesive have an impact on the effective transfer length (see Section 4.2), which may degrade over time due to environmental factors (i.e., moisture, UV from sunlight) or release chemicals that are not permitted in some applications (e.g., structures for processing drinking water). Furthermore, surface installation with adhesives may require preparation of the substrate surface, typically cleaning off dust and grease, removal of the external layer that might not exhibit sufficient cohesion, and potential reduction of the surface roughness by sandblasting or mechanical grinding the areas that may be too smooth or levelling of the cavities with suitable putty compounds (see Figure 7a). Depending on the installation conditions, it could be necessary to pre-install the sensing cable in the desired position with temporary fasteners before applying the adhesive in order to ensure that it will remain in position during curing of the adhesive (see Figure 7b).

The most common types of adhesives used for sensing cable installation are epoxy, cyanoacrylate, acrylic, and silicone sealants, which exhibit different Young’s moduli (from 3.5 GPa for epoxy to approximately 200 Mpa for acrylic and silicone sealant) and resistance to temperature (cyanoacrylate can be used only up to 80 °C, while the other types of adhesives can withstand temperatures exceeding 100 °C).

Sensors installed with special removable clamps have been reported in the literature [34] for specific applications (railway monitoring) where the substrate needs to be inspected or restored to pristine conditions without traces of glue.

#### 4.1.2. Embedding in Concrete

**Figure 8:**
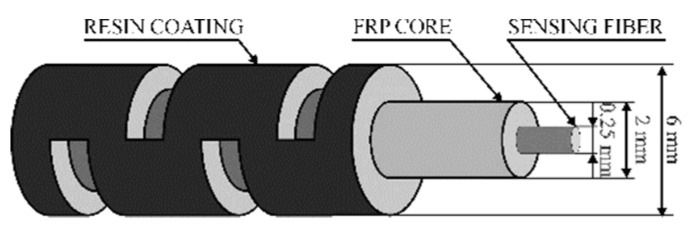
Example of a strain sensing cable with a structured surface designed for embedding in concrete elements.

Laying down of the sensing cable before the casting of concrete members is a common and convenient method of installation that can be used for newly-built elements [25,35,36,37]. Usually, sensing cables for concrete embedding are characterized by a structured external surface to ensure a good mechanical interlocking. Figure 8 shows an example of such a cable. The cable must provide some moisture protection of the sensing fibers since Portland-based mortars require some curing time at very high humidity; however, in most situations, moisture is not a significant threat after curing. Applications where concrete elements remain in contact with water (i.e., dams, tunnels) are an exception and require cables with increased moisture protection (i.e., fiber in metal tube (FIMT)).

The concrete casting process might compromise the cable integrity due to the drag effect that the material being poured can apply to the cable, and due to the possible impact of the aggregates that may have dimensions up to several centimeters and that may have sharp edges. A common practice consists of installing the sensing cable to the lower face of the steel rebar, i.e., using cable ties, before the concrete casting phase (see, for example, Barrias et al. [36]). In particular, deployment of the cable under the rebar ensures good protection against the possible impact of aggregates during the casting phase. It is also important to plan the ingress and egress of the sensing cable properly to minimize the risk of damage during the activities following the concrete casting.

**Figure 9:**
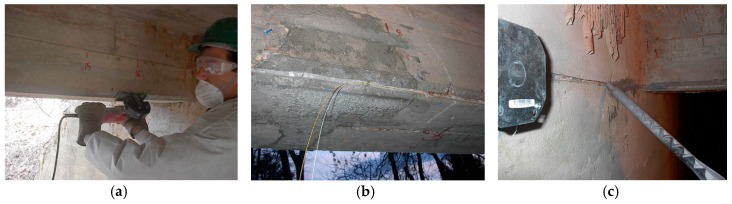
Near-to-surface installation: (**a**) groove milling, (**b**) cable installation, and (**c**) groove filling.

Near-to-surface (NSF) installation is a special procedure consisting of the deployment of the sensing cable inside grooves that are milled on the surface of the substrate and then filled with mortar or a suitable putty compound [38]. This technique, depicted in Figure 9, has the advantage of providing very effective protection of the sensing fibers during all the activities following installation of the sensing cable.

However, in terms of installation cost and time, the NSF technique may be slightly more demanding than other surface bonding techniques; hence, it is generally preferable when potentially dangerous work activities might occur in the surrounding area (i.e., welding of armors, moving of heavy objects).

#### 4.1.3. Embedding in Soil

A variety of interesting strain-sensing and leak-detection geological applications, such as the monitoring of landslides, embankments, levees, channels, shore erosion, etc., requires embedding the sensing cable into the soil [39,40,41,42]. The effective attachment of the sensor to the surrounding earth mass is recognized [43] as the main critical point in order to obtain reliable distributed measurements. In the literature, many attempts characterized by direct embedding of the sensing fibers in the soil are reported [44,45,46,47], as well as studies on the interaction between the soil and sensing fiber [6,7,8,9]. The experiments performed under plane-strain conditions highlight a good agreement between the strain distribution pattern measured by D-FOS with that measured using other measurement techniques (i.e., photogrammetry and particle image velocimetry (PIV)). However, the strain magnitude is much smaller due to low deformation compatibility between the optical sensing cable and soil material [48,49,50,51]. The reliability of the soil strain measurement obtained with D-FOS is fully conditioned by the cable-soil interface failure, which depends heavily on the type of soil [10], as well as on its density and water content. As a result, the reliability also changes during the lifetime of the sensing system.

The cable–soil interface behavior can be measured in terms of the pull-out force versus displacement [48], and in the absence of anchors or drag elements, can be modelled [52] according to three subsequent coupling phases:

(15) $$\begin{array}{c} (\mathrm{Phase}\;I)\;P\;=\;\frac{-4DG}{\beta h}\tanh\left(\beta L\right)u_{0},\\ (\mathrm{Phase}\;\mathrm{II})\;P\;=\;\frac{-AE}{L_{p}}\left(u_{0}\;+\;\frac{\tau_{max}h}{2G}\right)\;+\text{ D}L_{p}\tau_{max},\;(\mathrm{Phase}\;\mathrm{III})\;P\;=\;-2DL\tau_{max}, \end{array}$$

where *P* is the pull-out force, *L_p_* is the length of the plastic zone, *D* is the cable diameter, *E* is the cable Young’s modulus, *L* is the embedded length, *G* is the cable-soil shear modulus, *τ_max_* is the shear debonding stress, *h* is the thickness of the shearing area around the cable, and *β* is given by:

(16) $$\beta =\sqrt{\frac{4DG}{EAh}}.$$

**Figure 10:**
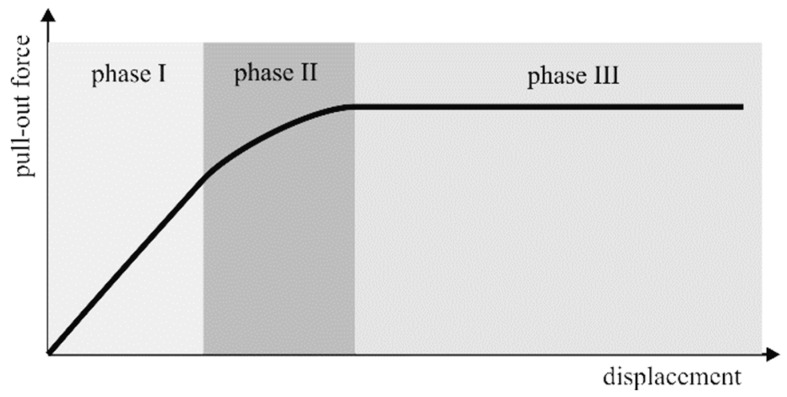
Analytical model phases of the soil–cable interface behavior.

**Figure 11:**
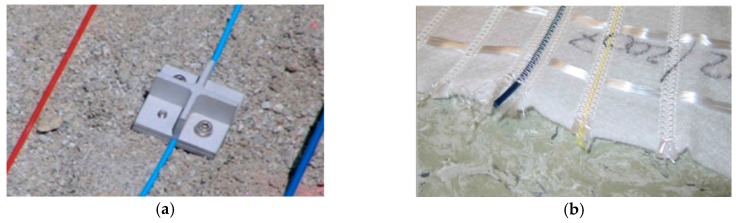
Example of (**a**) an anchor (Marmota Engineering AG, Zurich, Switzerland) and (**b**) a smart geotextile (STFI, Chemnitz, Germany).

The three phases are illustrated in Figure 10: initially, for a low pulling force, the friction coupling is fully effective and a pure elastic behavior is observed (phase I); when the force is increased, the coupling partially fails at some point, originating an elasto-plastic behavior (phase II); finally, at an even higher force, the decoupling is total and a pure plastic behavior is observed (phase III). Due to the fact that the reliability of the soil–strain measurement starts to degrade outside of phase I, it is of interest to extend this phase by increasing the shear debonding stress threshold (*τ_max_*) with anchor [53] (Figure 11a) or drag elements fixed to the cable and intended to extend the interface area between the sensor and the soil to provide some mechanical interlocking and dragging effects that could move the cable together with the flow of the soil aggregates. Smart geotextiles consisting of sensing cables mechanically coupled to geotextiles [54,55,56,57] or geo-nets [58] represent a particularly interesting case of drag-enhanced sensors that can increase the reliability of the measurement, even in soils characterized by extremely weak aggregation; in some cases, they can also provide additional functions, such as stabilization or leaching/washout reduction. An example of a smart geotextile is reported in Figure 11b.

#### 4.1.4. Embedding in Composites

**Figure 12:**
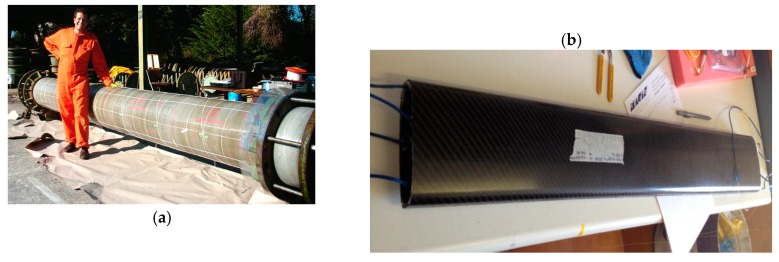
Examples of smart composite components with embedded fiber optic sensors: (**a**) glass-fiber reinforced polymer pipe and (**b**) carbon-fiber reinforced polymer sailboat mast section.

Embedding of fiber optic sensors in composite materials is extremely attractive in terms of creating smart structural components with an intrinsic sensing capability to be used in a huge variety of applications [59] in different sectors, such as the aerospace [60], energy [61], and marine [62] sectors. Figure 12 reports two examples of the integration of fiber optic sensors in composite components.

Integrating fiber optic sensors in composite elements generally requires implementation during manufacturing of the component itself and normally involves the use of fibers with special coatings, such as polyimide, that can withstand the elevated temperatures and pressures of most composite manufacturing processes. Compression of the sensing fiber between the different layers of the fabric structure may become a strong source of microbending attenuation, where this has especially been observed [63,64,65] at the points where the fiber enters and exits the composite material, hence requiring special attention and suitable technological solutions [60]. When implementing protecting devices, such as plastic tubes or embedded connectors, care must be taken to minimize the potential reduction in the strength of the composite.

Another possible cause of strength reduction in the composite is due to the mismatch between the sensing and composite fibers’ dimensions. However, the use of small-diameter fibers [66] or the embedding of standard fibers parallel to the reinforcing fibers [67] have been proven to mitigate this issue.

### 4.2. Strain Transfer

Whatever the fiber layout, embedded or surface-bonded, the intermediate layers absorb a portion of the strain present in the host material with the result that only a percentage of the strain is effectively sensed by the optical fiber. Over the last three decades, a multitude of researchers have recognized the crucial role of the strain transfer and have developed several mathematical models to predict the behavior and the performance of different types of optical fibers. Most of these models were based on the shear lag theory, first introduced by Cox in 1952 [68], which hypothesizes that the matrix bears only shear loads. In 1989, Claus et al. discussed the main advantages of embedding optical fibers into materials [69]. The embedding process was analyzed, leading to important considerations regarding the strain transfer. The fiber-to-matrix interface was identified as a key region. In particular, it was highlighted that voids and cracks, generated during the manufacturing process may alter the strain transfer performance. Additionally, it was noted how the mechanical properties of the fiber coating could influence the sensing capability of the optical fiber. In 1991, Nanni et al. considered the use of fiber optic sensors for the diagnostics of concrete structures [70]. The researchers embedded the optical fibers inside concrete cylinders produced in their laboratory. The specimens were tested under uniaxial compression and tension loads with the fibers were positioned perpendicularly and parallel with respect the cylinder axis. The authors discussed fiber orientation and embedding effects on the strain transfer process, confirming the high potential of using optical fibers for the monitoring of concrete structures. In 1992, Pak analyzed the strain transfer of a coated optical fiber embedded in an isotropic homogeneous matrix under a far-field longitudinal shear stress [71]. The study contributed remarkably to addressing the effects of varying the ratio between the shear modulus of the coating and the shear modulus of the host material. It was found that when this ratio is lower than one, a thinner layer of coating material produces a greater strain transfer. Conversely, when the ratio is above one, the effect is exactly the opposite. Finally, the optimal value for the coating shear modulus in order to obtain an optimum shear transfer was found to be equal to the geometric mean of the shear moduli of the fiber and the host material. Six years later, in 1998, Ansari and Libo developed a mathematical model for an optical fiber embedded in a host material with three concentric layers [72]. The research focused on the ability of the sensor to detect the strain distribution in the surrounding structure. The level of strain loss caused by the presence of the coating layer was quantified and a mathematical model was developed, which the researchers validated through a series of experiments. The theory retains its validity if three main assumptions are satisfied:

A1. All the materials are considered to operate within the elastic range.
A2. The interface between each pair of layers can be considered ideal, meaning that local flaws can be ignored and no debonding can occur.
A3. It is assumed that the core and cladding of the optical fiber are characterized by the same mechanical properties, behaving as a single layer of material referred to as the fiber core.

**Figure 13:**
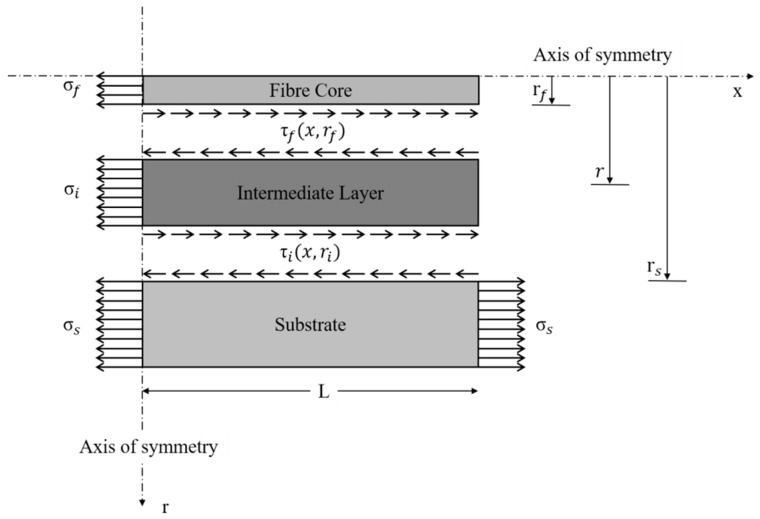
Free body diagram of the mechanical model.

Figure 13 represents a schematic view of the mechanical model, the development of which starts from the equilibrium of a fiber coating segment, here denoted as the intermediate layer along the x-direction:

(17) $$2\pi r_{f}\int_{0}^{L}\tau_{f}\left(x,r_{f}\right)dx\;+\;\pi \left(r_{i}^{2}\;-\;r_{f}^{2}\right)\sigma_{i}\;-\;2\pi r_{i}\int_{0}^{L}\tau_{i}\left(x,r_{i}\right)dx\;=\;0.$$

If the gauge length is much higher than the fiber radius ($L\;\gg \;r_{i}^{2}\;-\;r_{f}^{2}$), it is possible to reformulate Equation (17) as follows:

(18) $$\tau_{i}\left(x,r_{i}\right)\;=\;\frac{r_{f}}{r_{i}}\tau_{f}\left(x,r_{f}\right).$$

Exploiting Hooke’s law, it is possible to write another expression for $\tau_{i}$, as follows:

(19) $$\tau_{i}\left(x,r_{i}\right)\;=\;G_{i}\gamma_{i}\left(x,r_{i}\right),$$

where $\gamma_{i}\left(x,r_{i}\right)$ and $G_{i}$ represent the shear strain and the shear modulus of the intermediate layer, respectively.

**Figure 14:**
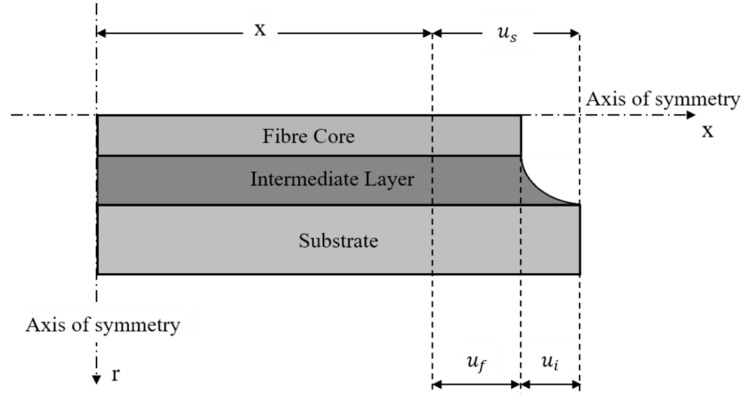
Schematic showing the compatibility conditions.

The model is developed by imposing no axial load at the extremities of the cable, thereby considering the host material as the only component subjected to external forces. Referring to Figure 14, the analysis continues by introducing the compatibility condition linking the axial displacement of the substrate material $u_{s}$ with the axial displacement of the fiber core $u_{f}$ and the shear deformation of the coating $u_{i}$:

(20) $$u_{s}\left(x\right)\;=\;u_{f}\left(x\right)\;+\;u_{i}\left(x\right).$$

In order to close the problem, the strategy is to find each of the three terms of Equation (20), where the left-hand side, $u_{m}\left(x\right)$, can be written as:

(21) $$u_{s}\left(x\right)\;=\;\int_{0}^{x}\varepsilon_{s}\left(x^{\prime }\right)dx^{\prime }\;=\;\int_{0}^{x}\frac{\sigma_{s}\left(x^{\prime }\right)}{E_{s}}dx^{\prime },$$

with $\varepsilon_{s}$, $\sigma_{s}$, and $E_{s}$ being the normal strain, stress, and Young’s modulus of the substrate material, respectively. Analogously, the fiber core displacement, $u_{f}\left(x\right)$, can be addressed as:

(22) $$u_{f}\left(x\right)\;=\;\int_{0}^{x}\varepsilon_{f}\left(x^{\prime }\right)dx^{\prime }\;=\;\frac{1}{\pi r_{f}^{2}E_{f}}\int_{0}^{x}\frac{T_{f}\left(x^{\prime }\right)}{E_{s}}dx^{\prime }.$$

In Equation (22), $\varepsilon_{f}$ and $E_{f}$ represent the normal strain and the Young’s modulus of the fiber core, respectively. Furthermore, the term $T_{f}$ symbolises the axial tensile force along the core of the fiber and can be found using the following expression:

(23) $$T_{f}\left(x\right)\;=\;\pi r_{f}^{2}\sigma_{f}\;-\;2\pi r_{f}\int_{0}^{x}\tau_{f}\left(x^{\prime },r_{f}\right)dx^{\prime }.$$

Finally, the last term of Equation (20), $u_{i}\left(x\right)$, is found by exploiting a combination of Equations (18) and (19), together with the small deformation assumption stating that:

(24) $$\gamma_{i}\left(x,r_{i}\right)\;=\;\frac{dx}{dr}.$$

Therefore, $u_{i}\left(x\right)$ is given by:

(25) $$u_{i}\left(x\right)\;=\;\int_{r_{f}\;}^{r_{s}}\frac{\tau_{f}\left(x,r_{f}\right)}{G_{f}}\frac{r_{f}}{r}dr\;=\;\frac{r_{f}}{G_{f}}\tau_{f}\left(x,r_{f}\right)\ln\frac{r_{s}}{r_{f}}.$$

Substituting these expressions of $u_{s}$, $u_{f}$, and $u_{i}$ into the compatibility condition expressed by Equation (20), and differentiating twice, leads to Equation (26):

(26) $$\tau_{f}^{\prime \prime }\left(x,r_{f}\right)\;-\;k^{2}\tau_{f}^{\prime }\left(x,r_{f}\right)\;=\;0,$$

where *k* is defined as the shear lag parameter with the following expression:

(27) $$k\;=\;\sqrt{\frac{2G_{i}}{r_{f}^{2}E_{f}\ln\left(\frac{r_{s}}{r_{f}}\right)}}.$$

Equation (26) is a second-order differential equation and needs two boundary conditions to determine the two parameters appearing in its general form solution. These two boundary conditions are represented by:

(28) $$T_{f}\left(0\right)\;=\;\pi r_{f}^{2}\sigma_{f},$$

(29) $$T_{f}\left(L\right)\;=\;0.$$

Physically, the first boundary condition translates to imposing that in a fiber with length equal to 2*L*, the strain measured by the fiber corresponds exactly to the strain of the substrate material at the axis of symmetry. Instead, the second boundary condition simply states that, at the extremities of the fiber, the axial force at the core is null.

Finally, the strain transfer model is summarized in:

(30) $$\varepsilon_{f}\left(x\right)\;=\;\frac{\sigma_{s}}{E_{s}}\left[1\;-\;\frac{\sinh\left(kx\right)}{\sinh\left(kL\right)}\right].$$

Another strain transfer model that is worth mentioning is that developed by Li et al. in 2006 [73]. This approach utilizes the same three main assumptions of Ansari’s model. Moreover, it considers a three-layered configuration such that Figure 13 and Figure 14 still describe the problem. Again, the starting point is to consider the stress equilibrium for a small fiber segment. Although the mathematical steps are slightly different with respect the aforementioned model, the conclusions are similar. As opposed to Equation (26), in which the unknown is the shear stress, here the authors directly derived a second-order differential equation for the axial fiber strain:

(31) $$\varepsilon_{f}^{\prime \prime }\left(x\right)\;-\;k^{2}\varepsilon_{f}\left(x\right)\;-\;k^{2}\varepsilon_{s}\;=\;0,$$

where the expression of *k* given by Equation (27) is still valid.

The general solution for Equation (31) is represented by Equation (32):

(32) $$\varepsilon_{f}\left(x\right)\;=\;c_{1}e^{kx}\;-\;c_{2}e^{-kx}\;+\;\varepsilon_{s}\;=\;0.$$

The key difference between the Ansari and Li models are the applied boundary conditions. The first boundary condition is analogous to Equation (29), imposing no strain transfer at the two ends of the fiber, but the second does not state that at the axis of symmetry the strain has been completely transferred. Indeed, Li et al. remove this assumption and impose that at the midpoint, the shear stress is zero. This difference transforms Equation (30) into Equation (33):

(33) $$\varepsilon_{f}\left(x\right)\;=\;\varepsilon_{s}\left[1\;-\;\frac{\cosh\left(kx\right)}{\cosh\left(kL\right)}\right].$$

This last formulation proved to be more accurate than the former introduced by Ansari, finding good agreement with the conclusions derived in other studies [74,75].

From a practical point of view, it is interesting to compute the average strain transfer rate $\bar{\alpha }$. Indeed, the strain sensed by an optical fiber corresponds to the average strain along the fiber length. Therefore, the knowledge of $\bar{\alpha }$ could allow for a reconstruction of the actual strain, $\varepsilon_{s}$, of the substrate. The average axial strain can be defined as follows:

(34) $$\bar{\varepsilon_{f}}\;=\;\frac{1}{2L}\int_{0}^{L}\varepsilon_{f}\left(x\right)dx.$$

Therefore, the average strain transfer rate is obtained by dividing Equation (34) by the strain present in the host material $\varepsilon_{s}$:

(35) $$\bar{\alpha }\;=\;\frac{\bar{\varepsilon_{f}}}{\varepsilon_{s}}.$$

Substituting into Equation (35), the respective values of $\varepsilon_{f}$, the two models return:

(36) $$\bar{\alpha }\;=\;1\;-\;\frac{\cosh\left(kL\right)}{kL\sinh\left(kL\right)}$$

for Ansari et al. and

(37) $$\bar{\alpha }\;=\;1\;-\;\frac{\sinh\left(kL\right)}{kL\cosh\left(kL\right)}$$

for Li et al.

It is clear that the bonded length has a great impact on the sensor performance. Therefore, it is legitimate to wonder what minimum length should be considered when developing an optical fiber measuring system. Li et al. computed the critical adherence length, *l_c_*, by imposing that the strain transfer rate at the midpoint is greater than 0.9, which translates to solving the following equation with *l_c_* as an unknown:

(38) $$1\;-\;\frac{\cosh\left(0\right)}{\cosh\left(kl_{c}\right)}\;\geq \;0.9.$$

Although this definition is somewhat arbitrary and other formulations are available in the literature, it does give a first approximation for the minimum required bonded length.

In the same study, the authors extended the three-layered concentric model to a multi-layered concentric model. From a mathematical point of view, the only difference is the definition of the shear lag parameter *k*, which assumes a more complex expression:

(39) $$k^{2}\;=\;{\left\{\frac{1}{2}r_{f}^{2}E_{f}\left[\overset{n}{\underset{i\;=\;2}{\sum }}\frac{1}{G_{i}}\ln\left(\frac{r_{i}}{r_{i\;-\;1}\;}\right)\;+\;\frac{1}{G_{1}}\ln\left(\frac{r_{1}}{r_{f}\;}\right)\right]\right\}}^{-1},$$

where *i* denotes the *i*^th^ layer and *n* denotes the number of layers in the optical fiber layout.

The shear lag parameter plays a key role in the strain transfer process because $\varepsilon_{f}$, $\bar{\varepsilon_{f}}$, and $l_{c}$ strictly depend on *k*. It condenses the mechanical (i.e., material stiffness) and geometrical properties (i.e., layers’ layout) of the sensing cable. The higher its value, the faster the strain transfer mechanism between two layers; therefore, it can be used to predict the performance of the sensing system.

In 2009, a refined formulation for the shear lag parameter for a concentric layer structure was proposed by Li et al. by considering the influence of the fiber on the host material, which has been neglected in all previous studies [76].

The resulting expression is given by Equation (40), while the strain transfer formulation of Equation (33) remains valid:

(40) $$k\;=\;\sqrt{\frac{2}{r_{f}^{2}E_{f}\left\{\frac{1}{G_{i}}\ln\left(\frac{r_{i}}{r_{f}}\right)\;+\;\frac{1}{G_{s}}\left[\frac{r_{s}^{2}}{r_{s}^{2}\;-\;r_{i}^{2}}\ln\left(\frac{r_{s}}{r_{i}}\right)\;-\;0.5\right]\right\}}}.$$

Here, in comparison with Equation (27), *k* also depends on the substrate material shear modulus and the radius of the intermediate layer. Analogously, the same expression related to a multi-layered concentric model can be derived as follows:

(41) $$k^{2}\;=\;\frac{2}{r_{f}^{2}E_{f}\left\{\frac{1}{G_{1}}\ln\left(\frac{r_{1}}{r_{f}}\right)\;+\;\sum_{i\;=\;2}^{n}\frac{1}{G_{i}}\ln\left(\frac{r_{i}}{r_{i-1}\;}\right)\;+\;\frac{1}{G_{s}}\left[\frac{r_{s}^{2}}{r_{s}^{2}\;-\;r_{n}^{2}}\ln\left(\frac{r_{s}}{r_{n}}\right)\;-\;0.5\right]\right\}}.$$

In all the previously considered models, the fiber was embedded in a host material such that a concentric layer model was suitable to describe the physics behind the problem. However, in most cases, optical fibers are surface-bonded rather than embedded. Thus, further protective layers are required to ensure a good isolation from corrosive agents and from possible damage produced by the mechanical coupling with the host material [77]. Dealing with surface-bonded rather than embedded optical fibers complicates the scenario because of the asymmetric configuration that is created [78]. In many cases, asymmetries arise from the cable geometry itself, of which the cross-section deviates from the traditional circular shape. In those circumstances, theoretical analyses are arduous to apply, and the predicted results may deviate from experimental data. Therefore, researchers are forced to rely on experiments and numerical simulations, such as finite element method (FEM) analyses.

**Figure 15:**
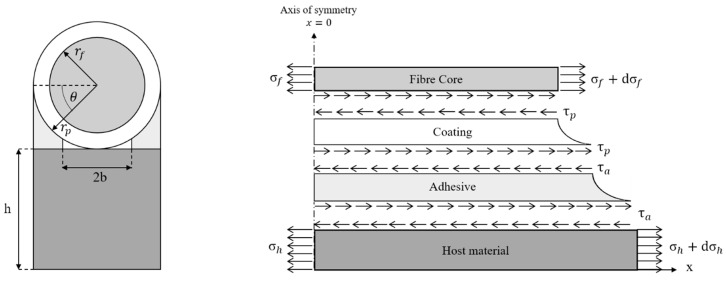
Analytical model of the surface-bonded optical fiber.

In 2011, Her and Huang developed a strain transfer model for a surface-bonded optical fiber [79]. The layout is represented in Figure 15. It is a four-layered model with the adhesive being filled between the host material and the protective coating. Moreover, the possibility of a small gap with width equal to 2b between the cable and substrate is considered in order to recreate a more realistic scenario.

There are four main hypotheses behind the development of this model. Three of them are the same of the Ansari’s theory. The fourth assumption states that the protective coating, as well as the adhesive, are subjected only to shear deformations, which is justified by the fact that their stiffness is significantly lower than the one related to the host material and the optical fiber. Starting from the equilibrium of a coating segment, the author develops a new formulation for the shear lag parameter, which now is defined as follows:

(42) $$k\;=\;\sqrt{\frac{2r_{p}}{\pi r_{f}^{2}}\left(\frac{\pi r_{f}^{2}}{2hr_{p}E_{h}}\;+\;\frac{1}{E_{f}}\right)\overset{\cos^{-1}\frac{b}{r_{p}}}{\underset{0}{\int }}\frac{1}{\frac{r_{p}\left(1\;-\;\sin\theta \right)}{G_{a}}\;+\;\frac{r_{p}}{G_{p}}\ln\frac{r_{p}}{r_{f}}}}d\theta ,$$

where $E_{h}$ and $E_{f}$ are the Young’s moduli of the host material and the fiber, respectively.

Analogously, $G_{a}$ and $G_{p}$ represent the shear moduli of the adhesive and the protective coating. Similar to the previous models, a second-order differential equation to be solved by applying appropriate boundary conditions is obtained. Here, at the ends of the bonded region, the following conditions are imposed:

(43) $$\{\begin{array}{c} \sigma_{f}\left(-L\right)\;=\;0,\\ \sigma_{f}\left(+L\right)\;=\;0. \end{array}$$

To summarize, the strain induced in the optical fiber core due to the stress state of the host material is given by Equation (44):

(44) $$\varepsilon_{f}\left(x\right)\;=\;\frac{\varepsilon_{0}}{\frac{\pi r_{f}^{2}}{2hr_{p}E_{h}}\;+\;\frac{1}{E_{f}}}\left[1\;-\;\frac{\cosh\left(kx\right)}{\cosh\left(kL\right)}\right],$$

where $\varepsilon_{0}$ is the far-field strain in the host material. The phenomenon is governed by hyperbolic cosine functions, highlighting a clear similarity with the previous theoretical results. Experimental results have been used to validate this model, which was shown to be able to take into account the effects of all the considered variables related to the material and the geometry.

**Figure 16:**
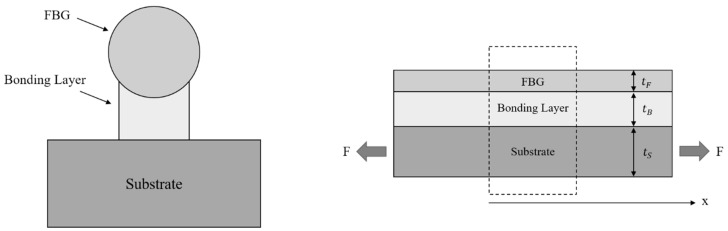
Geometry layout and free-body diagram for a surface-bonded fiber Bragg grating (FBG).

An alternative approach for surface-bonded fibers was proposed by Li et al. in 2009 [80]. A novel strain-transfer model was developed and validated numerically and experimentally by means of fiber Bragg grating (FBG) sensors. It is legitimate to ask whether the use of non-distributed-based measurements is suitable for this type of analysis. Nevertheless, the strain-transfer mechanism per se is independent from the fiber type and sensing physical principle because it is a purely structural problem. Therefore, FBG sensors can be used to test and validate new strain transfer theories, while being aware that, in case of strain gradients, only the average strain over the gauge length of the specific sensor deployed will be sensed. The assumptions A1, A2, and A3 are implied in the theory development, and the geometry layout is described in Figure 16, which considers an infinitesimal fiber segment.

The authors defined non-dimensional variables as follows:

(45) $$\bar{x}\;=\;\frac{x}{L},\;{\bar{k}}^{2}\;=\;\frac{GL^{2}}{E_{F}t_{F}t_{B}}\left(1\;+\;\frac{1}{\varphi }\right),\;\varphi \;=\;\frac{t_{S}E_{S}}{t_{F}E_{F}},$$

where *L* is the bounding length, *G* is the shear modulus of the bonding layer, and *E* is the Young’s modulus of the considered materials.

For the first time, a formulation is given, not only for the fiber core, but also for the substrate material, highlighting the influence of the fiber in the substrate strain field, especially if it is thin and with a low Young’s modulus. The mathematical derivation of Equation (46) was provided in a previous publication from the same authors [81]:

(46) $$\{\begin{array}{c} \varepsilon_{S}\left(\bar{x}\right)\;=\;\frac{\varphi }{\varphi \;+\;1}\varepsilon_{S}^{+}\;+\;\frac{\cosh\bar{k}\bar{x}}{\left(\varphi \;+\;1\right)\cosh\bar{k}}\varepsilon_{S}^{+}\\ \varepsilon_{F}\left(\bar{x}\right)\;=\;\frac{\varphi }{\varphi \;+\;1}\varepsilon_{S}^{+}\;-\;\frac{\varphi \cosh\bar{k}\bar{x}}{\left(\varphi \;+\;1\right)\cosh\bar{k}}\varepsilon_{S}^{+} \end{array},$$

with $\varepsilon_{S}^{+}$ indicating the substrate strain field at $\bar{x}\;=\;1$. Substituting $\bar{x}\;=\;0$ into the fiber-strain relation of Equation (28), the maximum strain transfer occurring at the midpoint is found to be:

(47) $$\varepsilon_{F}\left(0\right)\;=\;\frac{\varphi }{\varphi \;+\;1}\left(1\;-\;\frac{1}{\cosh\bar{k}}\right)\varepsilon_{S}^{+}.$$

The point is that $\varepsilon_{S}^{+}$ is not equal to the true strain of the substrate $\varepsilon_{T}$, which conceptually represents the strain in the host material without the surface-bonded FBG. Equation (48) returns the relation between $\varepsilon_{S}^{+}$ and $\varepsilon_{T}$:

(48) $$\varepsilon_{S}^{+}\;=\;\frac{\varphi }{\varphi \;+\;1}\varepsilon_{T}.$$

Then, substituting $\varepsilon_{S}^{+}$ into Equation (47), recalling the value of φ, performing some algebraic manipulations, and considering the cross-sectional areas of the substrate $A_{S}$, of the fiber $A_{F}$, and of the bonding layer $A_{B}$, the strain transfer relation is given by:

(49) $$\varepsilon_{F}\;=\;\left(1\;-\;\frac{1}{\cosh\bar{k}}\right)\left(\frac{t_{S}E_{S}}{t_{S}E_{S}\;+\;t_{F}E_{F}}\right)\left(\frac{A_{S}E_{S}}{A_{S}E_{S}\;+\;A_{F}E_{F}\;+\;A_{B}E_{B}}\right),$$

which is a comprehensive formula that considers the material and geometrical properties of all the considered layers in the analytical model. The model was proved to be consistent with FEM and experimental results performed in the same study and in a recent research work [82] where it was applied to a more complex fiber–substrate configuration.

### 4.3. Crack Detection Capability

**Figure 17:**
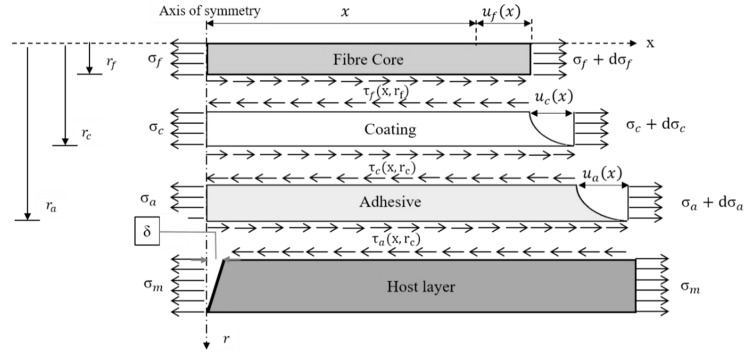
Feng’s model with crack opening displacement (COD) = 2δ.

The strain transfer phenomenon is often perceived to be a problem because the fiber is not sensing the actual strain in the host material. The situation is different when dealing with the detection of crack opening displacements (CODs). Indeed, the spreading effect due to the occurring shear lag allows one to sense a COD, even at a certain distance, and may prevent the failure of the fiber core. In recent years, attention has shifted to the study of this phenomenon. Figure 17 illustrates the model proposed by Feng et al. in 2013, which is applied to D-FOS [83].

It is a four-layered model with a COD equal to 2δ based on three assumptions. The first regards the material behavior of the four layers. In the previous models all the materials were treated in a liner elastic manner (A1). Here, all the materials operate inside the elastic range, but the polymeric coating layer is considered to be an ideal elastoplastic material with the following constitutive law:

(50) $$\tau_{c}\;=\;\{\begin{array}{ll} G_{c}\gamma_{c} & \gamma_{c}\;=\;\frac{\tau_{cr}}{G_{c}}\\ \tau_{cr} & \gamma_{c}\;\geq \;\frac{\tau_{cr}}{G_{c}} \end{array}.$$

The second assumption is analogous to A2, implying that no debonding occurs at all at the interfaces while ignoring possible local imperfections. The third hypothesis regards the crack-induced strain field. In particular, it is assumed that such discontinuity in the material generates a strain concentration only in the proximity of the crack. Therefore, it is sufficient to analyze a small segment of the cable whose extension is at least one spatial resolution of the D-FOS.

The theory is developed by considering the compatibility conditions at an arbitrary fiber section:

(51) $$u_{m}\left(x\right)\;=\;u_{f}\left(x\right)\;+\;u_{c}\left(x\right)\;+\;u_{a}\left(x\right),$$

where $u_{m}\left(x\right)$ and $u_{f}\left(x\right)$ represent the deformations of the host layer and the fiber core, respectively. Furthermore, $u_{c}\left(x\right)$ symbolises the shear deformations of the coating, whereas $u_{a}\left(x\right)$ is the induced shear deformation in the adhesive. Equation (52) rewrites $u_{m}\left(x\right)$ as a function of the COD:

(52) $$u_{m}\left(x\right)\;=\;\varepsilon_{m}x\;+\;\delta .$$

Substituting Equation (52) into the compatibility conditions, and computing the remaining terms $u_{c}\left(x\right)$ and $u_{a}\left(x\right)$ by means of equilibrium relationships at a generic fiber section, it is possible to manipulate Equation (51) into a second order differential equation, with $u_{f}\left(x\right)$ as the only unknown:

(53) $$u_{f}^{\prime \prime }\;-\;k^{2}u_{f}\;=\;-k^{2}\left(\varepsilon_{m}x\;+\;\delta \right),$$

where *k* is the shear lag parameter defined as:

(54) $$k\;=\;\sqrt{\frac{2}{E_{f}r_{f}^{2}\left(\frac{1}{G_{c}}\ln\frac{r_{c}}{r_{f}}\;+\;\frac{1}{G_{a}}\ln\frac{r_{a}}{r_{c}}\right)}},$$

where $E_{f}$ represents the fiber core Young’s modulus, and $G_{c}$ and $G_{a}$ are the shear moduli of the polymeric coating and the adhesive, respectively.

Differentiating the general solution form of Equation (52) with respect to x returns the normal strain distribution along the fiber:

(55) $$\varepsilon_{f}\left(x\right)\;=\;-kc_{1}e^{k\left(L\;-\;x\right)}\;+\;c_{2}e^{k\left(L\;+\;x\right)}\;+\;\varepsilon_{m}\;=\;0.$$

The boundary conditions are derived by imposing a zero midpoint displacement of the fiber core, due to symmetry considerations, and assuming that the fiber strain at its ends equals the nominal uniform substrate material strain:

(56) $$\{\begin{array}{c} u_{f}\left(0\right)\;=\;0\\ \;\varepsilon_{f}\left(L\right)\;=\;\varepsilon_{m} \end{array}.$$

Hence the final strain transfer relation is given by:

(57) $$\varepsilon_{f}\left(x\right)\;=\;\varepsilon_{m}\;+\;\frac{k\delta \left(e^{k\left(3L\;-\;x\right)}\;-\;e^{k\left(L\;+\;x\right)}\right)}{e^{kL}\;+\;e^{3kL}}.$$

It is observed that $\varepsilon_{f}$ depends on the COD, the shear lag parameter (which condenses the geometrical and the mechanical properties of the considered layout), the position with respect the crack location, and the length of the glued D-FOS.

Finally, the author proposes an alternative formulation that considers possible plastic deformations of the coating layer in the case of large deformations. The mathematical description becomes complex, and for the sake of brevity, it is not reported here; nevertheless, the interested reader can find all the details in the paper of Feng et al. [83].

Other studies, such as the one made by Billon et al. in 2015, make use of exponential approximations for the strain profile [84]. The free body diagram of this model can be schematized again by Figure 17. Furthermore, the strain field generated in the fiber core due to the presence of a COD is formulated using a new approach:

(58) $$\varepsilon_{f}\left(x\right)\;=\;2\delta \cdot \mathrm{MTF}\left(x\right),$$

where k is again the shear lag parameter defined by Equation (54) and MTF is an acronym for “mechanical transfer function” and is given by:

(59) $$\mathrm{MTF}\left(x\right)\;=\;\frac{k}{2}\cdot e^{-k\left|x\right|}.$$

The model was in good agreement with experimental data and was also verified through numerical simulations. Equation (59) highlights how the strain spreading effect in the measured strain profile with a COD is inversely proportional to the shear lag parameter value, of which its key role in the strain transfer process is now well understood. In certain circumstances, the knowledge of the full width at half maximum (FWHM), related to the strain response after a COD, is useful for a comprehensive analysis. The FWHM is commonly defined as an expression of the spreading related to the peak of a function. In this case, it corresponds to the segment of the optical fiber that is carrying a strain value with a magnitude equal to or greater than half of the peak strain value in correspondence with the COD center. This definition applied to Equation (58) returns an expression for the FWHM that depends, as expected, on the shear lag parameter *k*:

(60) $$\mathrm{FWHM}\;=\;\frac{2\ln2}{k}.$$

The significance of the FWHM value emerges when a multiple crack state is present. Each crack that is present in the host material translates to a peak of strain at the fiber core. Therefore, when multiple cracks arise, it is expected that several strain peaks are observed, but this occurs only if these cracks are sufficiently far from each other. Indeed, below a critical distance, neighboring peaks merge together, complicating the analysis. Therefore, the FWHM parameter can be considered to be a measure of the strain spatial resolution because if the distance separating two cracks is lower than the FWHM value, then only one peak of strain can be sensed from the fiber. This consideration establishes a lower limit for the shear lag parameter *k*, depending on the minimum spatial resolution required in the design phase. Conversely, it has been reported in the literature [38] that excessively high *k* values could lead to a poor performance for Brillouin D-FOS. The presence of a COD produces a strain concentration in correspondence with the crack location. If the resolution limit of the interrogator equipment is insufficient, meaning that the strain is concentrated below the above-mentioned threshold, leakage in the Brillouin spectrum is observed due to a distance-averaging superposition. This condition degrades the interrogator performance and thus should be avoided. Spreading the strain concentration over a wider area leads to a smoother strain profile in the fiber core. This effect, despite being considered a drawback in multiple crack states, could be required to enhance the crack detection capability of Brillouin D-FOS. Therefore, this consideration establishes a higher limit for the shear lag parameter, depending on the spatial resolution limit of the Brillouin interrogator.

The use of analytical models as a tool for predicting the strain transfer is widely used in the literature. Nevertheless, their application is still limited to simple case studies [78]. Indeed, it is common to find cable jackets surrounding reinforcements rods and temperature compensation fibers. Therefore, the cross section may deviate from the traditional circular shape, making theoretical analyses arduous to apply. Researchers are forced to rely on experiments and numerical simulations, such as finite element method (FEM) analyses.

### 4.4. Nonlinear Stress Transfer and Overstrain Protection

All the analytical models considered were developed under the hypothesis of the linear elasticity of the materials involved. In a variety of practical applications, deformations above the linear elastic limit of the interface (or substrate) may be reached before reaching the ultimate strain of the sensing fiber (typically 3% for a SMF-28 fiber). In these cases, depending on the constitutive law of the stress-transfer interface, the strain profile that is transferred to the sensing fiber may be further distorted by fragile failure, plastic flow, or nonlinear viscoelasticity. This nonlinear behavior can be used to introduce some overstrain protection of the sensing fiber, and despite the enlargement of the measurement distortion, it must be considered as a beneficial key element for the cable design and appropriate cable selection.

**Figure 18:**
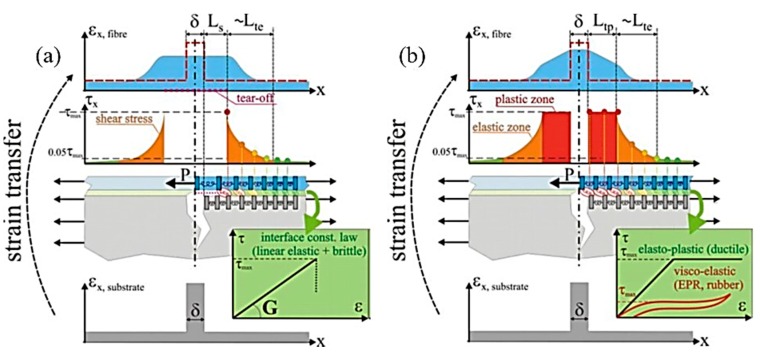
Strain transfer with rip-off slipping (**a**) and with ductile/rubber interfaces (**b**). EPR: ethylene propylene rubber.

Figure 18a schematically illustrates what happens when the core strength is above that of the interface (or of the substrate, whichever is lower). In this case, the point at which the peak shear stress equals the interface strength is reached before the failure of the sensing core, and this causes a tear-off (or rip-off for the substrate) propagation (of length 2*L_s_*) of the failure region, along which the sensing fiber slips with respect to the substrate. Along the whole slip length, the fiber will experience a flat strain profile of value *ε_f,peak_* = δ/(2*L_s_* + δ), which is much lower than the singularity peak strain on the substrate (COD). Any further increase of the substrate crack dδ causes a propagation of the failure slip length *L_s_*, but does not affect the strain in the sensing core, thus limiting the strain of the cable core below a maximum limit value. Similar considerations regarding this “fiber-protective slippage” can be found in the study of Imai and Feng [85]. This solution ensures a total overstrain protection of the sensing fibers but may introduce some measurement nonlinearity when recovering after the slip limit due to the frictions in the slipping of the fracture edges. If the substrate deformation is removed, the strain value *ε_f,peak_* on the detached length goes to zero. However, since in this area, the mechanical coupling between the substrate and sensing cable has been permanently removed, the fiber loses its capability to measure possible compressive deformations.

Figure 18b schematically illustrates what happens when the interface reaches a plastic ductile behavior at a yield point below the strength limit of the sensing core. In this case, when the peak shear stress reaches the yield point, a plastic zone of length *L_tp_* is created, along which the shear stress stabilizes around a constant value. Differently from Figure 18a, in Figure 18b, some stress transfer still occurs and the normal strain at the fiber core increases linearly until the total stress transferred to the fiber by the plasticized and elastic transfer lengths reaches the ultimate strength of the cable. Although this configuration does not provide a total overstrain protection, it has been reported by Feng et al. that the plastic deformation of the intermediate layers can improve the capacity of the fiber to withstand large deformations [83]. Indeed, the failure of the fiber core is delayed and will occur at higher COD values because the strain peak is significantly reduced. Since plastic deformations are permanent, even if the substrate deformation is recovered, this solution introduces a measurement offset when recovering after the plastic limit. Indeed, a residual “triangular peak” distortion is left on the sensed strain profile around the crack position. However, the mechanical coupling will still be present in the plasticized area, so if any compressive deformation is applied along this length, it will be sensed as a reduction of the “triangular peak.”

The case of nonlinear viscoelastic behavior is somehow similar to that of plastic ductile materials and it is also illustrated by the red line in the constitutive law diagram of Figure 18b.

The nonlinear viscoelastic behavior is typical of synthetic rubbers such as ethylene propylene (EPM) and ethylene propylene diene (EPDM). According to the ASTM nomenclature, the letter M at the end of the two acronyms symbolizes the “M class” and includes rubbers with a saturated chain of the polymethylene type [86].

EPM rubbers are binary copolymers, whereas EPDM rubbers are terpolymers where a third diene comonomer was included to enhance the peroxide crosslinking and to allow for sulfur vulcanization [87]. Despite their differences, both EPM and EPDM are commonly used as coating sheaths for commercial strain sensing cables and allow for elevated strain values. In their research, Klug et al. reported that the investigated sensing cable, made with a highly plastic EPM sheath and used for the monitoring of railway deformations, failed at strain values of the order of 8–9% [34]. The sensing fiber at the core of the cable was expected to fail for strain values at about 3%, thus demonstrating the beneficial effect of the EPM jacket. Although rubber-like materials allow for a quasi-complete recovery after huge strain levels [88], the possibility to observe residual deformations cannot be excluded. Indeed, they might exhibit a stress-strain hysteresis that, when the substrate deformation is decreased or removed, alter the original strain field, making it difficult to predict. Moreover, their stress–strain dependence may be very nonlinear and heavily dependent on temperature.

The viscoelastic response of rubber-like materials is tested using a dynamic mechanical analysis (DMA) approach. In DMA, a sinusoidal force is applied to the specimen and the stress-strain relationship is given by:

(61) $$\sigma \;=\;E^{*}\varepsilon ,$$

where $E^{*}$ represents the complex modulus of elasticity, which by definition is made of a real and an imaginary part, as follows:

(62) $$E^{*}\;=\;E^{\prime }\;+\;iE^{\prime \prime }.$$

**Figure 19:**
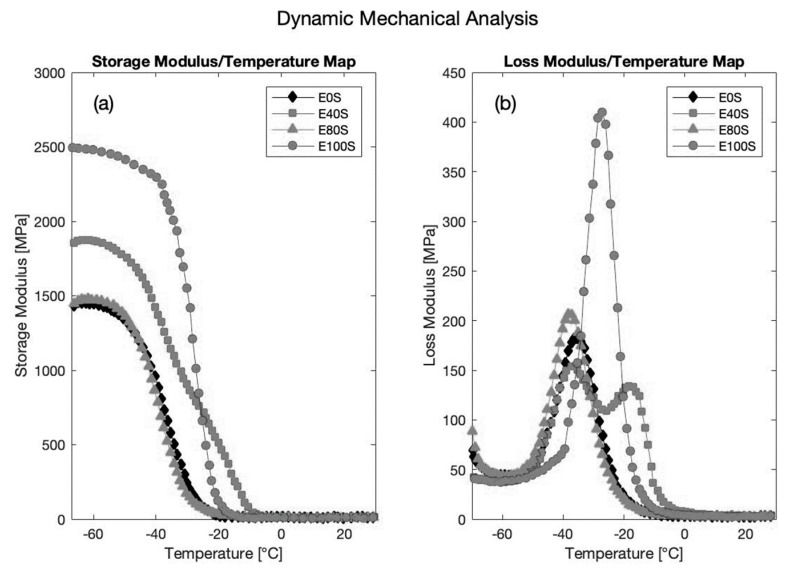
Effect of temperature on the storage modulus (**a**) and loss modulus (**b**) of sulfur-cured EPDM, SBR, and EPDM/SBR blends at a frequency of 10 Hz. Data collected from Nair et al. [90].

The real part $E^{\prime }$ is referred to as the *storage* modulus, whereas the imaginary component $E^{\prime \prime }$ is called the *loss* modulus. The former is a measure of the stored energy (elastic component) and the latter symbolizes the energy dissipated as heat (viscous component) [89]. In 2009, Nair et al. tested several EPDM and styrene-butadiene rubber (SBR) blends under different DMA conditions [90]. The legend of Figure 19 shows that several types of blends were tested using different percentages of EPDM and SBR (with E_100_S being pure EPDM and E_0_S being pure SBR). The “S” letter simply denotes the vulcanizing system sulfur. The results highlight the dependence of both the storage and loss moduli on the temperature at a given testing frequency of 10 Hz. The onset of the storage modulus drops or the peak of the loss modulus can be taken as references to determine the *glass transition temperature* T_g_, which identifies the glassy and rubbery regions [91].

Considering the above factors and the fact that the chosen testing frequency influences the T_g_ (the lower the frequency, the lower the glass transition temperature) [91], the viscoelastic response of EPM-EPDM coated cables is difficult to characterize and introduces huge measurement errors, especially at low and medium strain values. Therefore, cable sheaths made of viscoelastic materials seem to be suggested for those applications characterized by large strain levels but not requiring a high measurement accuracy.

The viscous behavior of rubbers is furthermore responsible for creep that slowly “flattens” the measured profile for a concentrated strain over time.

### 4.5. The Effect of Creep

**Figure 20:**
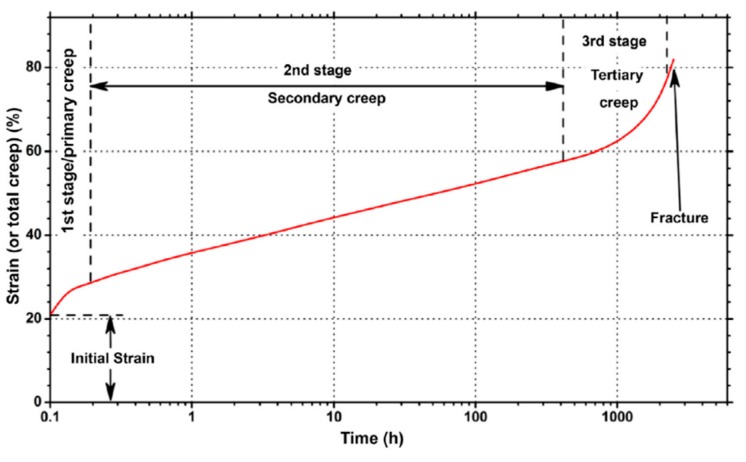
Example of creep behavior: strain versus time [94].

Creep, also known as “cold flow” in elastomer technology, is a plastic deformation that slowly increases in time, even if the material is subjected to stress levels below the yield strength of the material. Its phenomenological aspects can be characterized by performing a creep test, in which the time-dependent strain as a result of the application of a steady uniaxial stress is monitored [92]. Charting the data related to a creep test returns the plot shown in Figure 20, highlighting three different regions [93]. The first is referred to as primary creep and is characterized by a decreasing rate. The second stage, or secondary creep, can be considered to proceed at an almost constant rate. The last stage, or simply tertiary creep, shows an increasing strain rate and precedes fracture.

Creep can be considered negligible for metals at room temperature but must be taken into account when dealing with rubbers and soft polymers, such as EPM and EPDM, even at relatively low stress levels.

**Figure 21:**
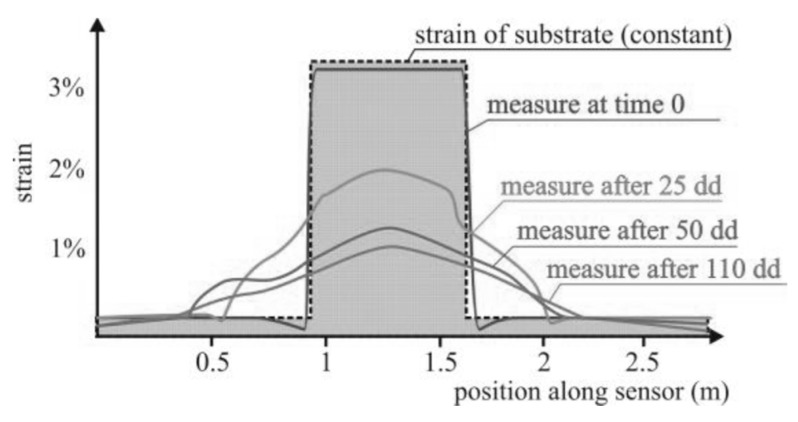
Example of strain profile spreading due to creep.

In the case of the sudden application of a concentrated strain in the host material, as usually happens with the cracking of concrete and stone-like materials, the creep in the strain transfer interface may cause a “spread” of the measured strain profile (Figure 21) that is progressive over time and may compromise the capability to correctly estimate the magnitude and location of the strain concentration. Creep spreading of the measured profile of a concentrated strain has been observed with cables having an ethylene propylene rubber (EPR) coating inside a controlled laboratory environment [34].

Under certain stress, strain, time, moisture, and temperature levels, most polymeric materials can be described with mathematical models based on a linear viscoelasticity. The limitations of this assumption depends on many different considerations and their estimation is not possible without an experimental campaign [93,95].

For materials having a linear creep response, the final strain after a certain amount of time is linearly proportional to the constant applied stress. Their creep behavior can be described by considering the “creep compliance” J(t) and the “stress relaxation modulus” [96]. The first is defined as the strain ϵ(t) to constant stress σ ratio:

(63) $$J\left(t\right)\;=\;\frac{\epsilon \left(t\right)}{\sigma },$$

whereas the latter is defined as the stress to constant strain ratio:

(64) $$G\left(t\right)\;=\;\frac{\sigma \left(t\right)}{\epsilon }.$$

Ji-long Li et al. built an ambitious mathematical model that aimed to predict the strain transfer phenomenon of an FBG fiber over a period of time by considering the effect of creep [97]. However, this theoretical model is of limited application since it requires an accurate knowledge of the creep compliances of the host material, of the adhesive, and of all the layers composing the optical fiber.

## 5. Conclusions

In this paper, a wide range of theoretical, analytical, and technological aspects concerning the design, development, and application of optical fiber cables for Brillouin-based distributed optical fiber sensors have been reviewed. It has been shown how the selection of type, coating, materials, and geometry of the fiber cable components can have a strong effect on the enhancement of the Brillouin signal; the sensitivity to the desired measured quantities, i.e., strain and temperature; and the effective protection from harsh environmental conditions over long distances and periods of time. Furthermore, the detection of defects, such as cracks, strongly depends on a suitable combination of both the sensing and cable system features. Further research is still needed to get a deeper insight into the strain transfer mechanisms, extending the models and results to different optical cables and installations.

All the considerations of the various aspects treated in the paper suggest that the fiber sensing cable plays an important role toward the effective implementation of Brillouin-based measurement techniques and therefore for their successful exploitation in many application fields. Regarding the technology readiness level, some commercial solutions are already present in the market and deployed in several sites for different monitoring or diagnostic purposes. However, standardization still requires additional work on the reliability of the measurement process in order to establish guidelines for the expression of uncertainty in the presence of the several influencing factors analyzed in this paper.
